# A multi-faceted intervention including antenatal corticosteroids to reduce neonatal mortality associated with preterm birth: a case study from the Guatemalan Western Highlands

**DOI:** 10.1186/s12978-016-0178-0

**Published:** 2016-05-24

**Authors:** Ana Garces, Elizabeth M. McClure, Lester Figueroa, Sayury Pineda, K. Michael Hambidge, Nancy F. Krebs, Vanessa R. Thorsten, Dennis D. Wallace, Fernando Althabe, Robert L. Goldenberg

**Affiliations:** FANCAP, Guatemala City, Guatemala; RTI International, Durham, NC USA; University of Colorado School of Medicine, Denver, CO USA; IECS, Buenos Aires, Argentina; University of Columbia School of Medicine, New York, NY USA

## Abstract

**Background:**

The Global Network for Women’s and Children’s Health Research undertook a cluster-randomized trial to assess the impact of a multi-faceted intervention to identify women at high-risk of preterm birth at all levels of care, to administer corticosteroids to women and refer for facility delivery compared with standard care. Of the seven sites that participated in the ACT trial, only two sites had statistically significant reductions in the neonatal mortality among the target group of <5th percentile infants, and of the two, Guatemala’s improvement in neonatal mortality was by far the largest.

**Methods:**

We used data available from the ACT trial as well as pretrial data in an attempt to understand why neonatal mortality may have decreased in the intervention clusters in <5^th^ percentile infants in Chimaltenango, Guatemala. The intervention and control clusters were compared in regards to ACS use, the various types of medical care, outcomes in facility and community births and among births in various birth weight categories.

**Results:**

Neonatal mortality decreased to a greater extent in the intervention compared to the control clusters in the <5^th^ percentile infants in Guatemala during the ACT Trial. ACS use for the <5^th^ percentile infants in the intervention clusters was 49.1 % compared to 13.8 % in the control clusters. Many measures of the quality of obstetric and neonatal care improved to a greater extent in the intervention compared to the control clusters during the trial. Births in facilities and births weighing 1500 to 2500 g had the greatest reduction in neonatal mortality.

**Conclusions:**

The combination of improved care and greater ACS use may potentially account for the observed difference in neonatal mortality between the intervention and control clusters.

**Trial registration:**

Clinicaltrials.gov: NCT01084096.

## Background

In high-resource countries, antenatal corticosteroids (ACS) given to the mother in the week prior to a preterm birth <34 weeks is associated with a reduction in respiratory distress syndrome, intraventricular hemorrhage, necrotizing enterocolitis and neonatal death [1–3]. Because few studies have evaluated the effectiveness of ACS in low-middle income countries (LMIC), from 2012 to 2014, the *Eunice Kennedy Shriver* National Institute of Child Health and Human Development (NICHD)’s Global Network for Women and Children’s Health Research conducted the Antenatal Corticosteroids Trial (ACT). This trial was carried out in Chimaltenango, Guatemala and 6 other sites in LMIC (Argentina, Kenya, Zambia, Pakistan and India [2 sites]). ACT was a cluster randomized trial which tested whether an intervention including birth attendant training and the provision of kits with ACS increased the use of ACS, and if use increased, whether the intervention was effective in reducing neonatal mortality and was safe [4]. Overall, the intervention substantially increased the use of ACS in the intervention clusters, but was not effective in reducing neonatal mortality in the targeted infants and was associated with a small but significant increase in overall neonatal mortality. However, two of the 7 sites had lower neonatal mortality in the targeted less than 5^th^ percentile infants in the intervention compared to the control clusters and of these two, the reduction in Guatemala was the greatest [5].

In Guatemala, the ACT study took place in the Chimaltenango region, an area in the Western highlands populated predominantly by indigenous peoples [6]. In that region, the infant mortality is generally higher than in other areas of Guatemala and has been associated with a high percentage of home births cared for by traditional birth attendants (TBAs) [7, 8]. For home births, pregnant women at risk for preterm birth rarely receive ACS. While ACS use is recommended by the Guatemala Ministry of Health (MOH), there are no data on the rate of use within hospitals.

In recent years, the Chimaltenango district hospital (Hospital Nacional de Chimaltenango), which serves as a referral hospital, has undergone extensive improvements. As a national law to improve maternal and neonatal care was implemented, there was a concerted effort by the MOH to improve the quality of care in the community health services and at this district hospital. The hospital, which in 2010 had five obstetricians and five pediatricians, as of 2015 had 11 obstetricians and 11 pediatricians on staff. There are residency programs in both obstetrics and gynecology and pediatrics, with 12 residents in each program. There is a newborn care nursery with intensive care and a referral system with ambulances available for transport from other lower level health services in the district. In addition, substantial effort has been directed to ensure that TBAs identify and make early referrals of pregnancies and deliveries, when needed, and to improve the overall collaboration between community level and institutional health personnel [8].

Thus, the Chimaltenango district hospital has many similar capabilities to those of the hospitals in high income countries, in which trials of ACS have shown benefit [3, 9–11].

Since 2010, in the Chimaltenango district, births inside a facility increased from one third to one half of the total and were accompanied by measurable improvements in the quality of care [12]. With these changes, there have been substantial improvements in both the neonatal mortality and stillbirth rates in the Chimaltenango area. It is in this setting that the ACT study took place from September 2012 to March 2014. The goal of this case study was to explore the potential reasons for the lower rate of neonatal mortality in the <5^th^ percentile infants in the intervention clusters compared to the control clusters in the Chimaltenango region of Guatemala during the ACT trial period.

## Methods

The NICHD Global Network undertook a cluster-randomized trial to assess the impact of a multi-faceted intervention to identify women at high-risk of preterm birth at all levels of care, to administer corticosteroids to women and refer for facility delivery compared with standard care. The methods for the ACT trial are described in detail elsewhere [4, 5], but briefly, at each site, intervention and control study clusters were included as part of a prospective Maternal Newborn Health Registry, in which study staff sought to identify all pregnant women and enroll them early in pregnancy and follow them through delivery [13, 14]. Clusters were geographically defined and generally included 300–500 pregnant women who delivered each year. Following randomization, the trial staff aimed to train all birth attendants within the intervention clusters on identifying signs of preterm birth, determining which mothers were within the gestational age range to be eligible for ACS, and transferring these mothers to a hospital to receive ACS.

In Guatemala, the trial occurred in 10 clusters in the Chimaltenango Province, located 60 km from Guatemala City, the capital of Guatemala. The MOH is the most common provider of health care in the region and physicians and nurses provide care through a network of ten health centers and 35 health posts. There is one single referral hospital for the region. In this intervention, preterm birth was identified at the community level and/or inside health services and women were referred to the Chimaltenango hospital for delivery and care.

### Outcomes

The outcome data were collected independently by trained Registry Administrators in a prospective Maternal and Newborn Health (MNH) registry [13, 14]. The primary outcome of the ACT trial was 28-day neonatal mortality among <5th percentile birth weight infants (proxy for preterm birth due to poor gestational age dating). The <5^th^ percentile was based on the pre-trial rates and the site-specific cut-off for Guatemala was 2,267 g. ACS administration and suspected maternal infection were secondary outcomes of the ACT trial. To define maternal infection, we collected data on clinical symptoms and process measures for a composite outcome of suspected infection (but did not have confirmed maternal infection).

We also explored the relationship between ACT treatment group and measures of delivery care, facility characteristics, and neonatal infection. Facilities were characterized as having cesarean section capabilities if two or more women received a cesarean section at the facility during the ACT trial period. Similarly, facility neonatal care capabilities including bag and mask, and oxygen or mechanical ventilation were determined in the same manner. For newborn infection, we used the Word Health Organization (WHO) Young Infants Clinical Signs Study criteria [15, 16] to define possible severe bacterial infection (PSBI). PSBI was defined as an infant with any of the following: breathing difficulty, feeding problems (i.e., stopped suckling or feeding), high fever (>38 °C), hypothermia (<35 °C), convulsions, and bleeding or pus-like discharge from umbilicus. Finally, we calculated 28-neonatal mortality rates stratified by delivery location and birth weight category.

### Statistical analyses

The ACT trial was powered to detect a 30 % reduction in 28-day neonatal mortality among <5th percentile birth weight infants but the trial was not designed to conduct country-specific analyses. A total of 10 defined geographic clusters in Guatemala were randomized within five randomization strata. Because one control cluster was dropped early in the trial due to security reasons which prevented study activities, we excluded both intervention and control data from that stratum. The trial period included births between September 2012 and March 2014 and used data for births occurring in 2010 as pretrial data.

Generalized linear models were used to evaluate the relationship between ACT treatment group and 28-day neonatal mortality and PSBI, and to develop point and interval estimates of relative risk (RR) associated with these risk factors. Additionally, we used a generalized linear model with an identity link to test whether the change in prevalence of specific prenatal and delivery characteristics from the pretrial to the trial periods differed by treatment group. For all models, generalized estimating equations were used to account for the correlation of outcomes within cluster to develop appropriate confidence intervals. Analyses were adjusted for randomization strata. Descriptive statistics (frequencies, percentages and rates per 1,000 live births) are provided for treatments, morbidities, and 28-day neonatal mortality. Analyses were performed by RTI International with SAS versions 9.3 and 9.4 (SAS Institute, Cary, NC, USA).

### Approvals

The trial was reviewed and approved by the ethics committees at each site, the World Health Organization and the NICHD. An independent data monitoring committee appointed by NICHD reviewed the progress of the trial, as specified in the protocol. All women provided informed consent prior to enrollment.

## Results

Table 1 presents the relative risk (RR) and 95 % confidence intervals (CI) for neonatal mortality in the <5^th^ percentile infants in the intervention compared to the control clusters during the ACT trial by site. Of the 7 sites that participated in the ACT trial, only 2 sites had significant reductions in 28 day neonatal mortality in the targeted group of <5^th^ percentile infants, and of the two, Guatemala’s improvement was by far the largest. The RR for neonatal death for <5th percentile infants in Guatemala in the intervention clusters was 0.74 (0.68, 0.81), *p* = <.0001. None of the sites, including Guatemala had an overall lower rate of neonatal mortality in the intervention clusters.

**Table 1:** Relative risk and 95 % confidence intervals for neonatal mortality by site for <5^th^ percentile births and all births

Research Site	<5^th^ %ile births RR (95 % CI)	All births RR (95 % CI)
Zambia	1.43 (0.90, 2.28)	1.77 (1.42, 2.20)
Kenya	1.30 (0.94, 1.81)	1.47 (1.02, 2.12)
Belgaum, India	0.96 (0.75, 1.22)	1.13 (0.99, 1.27)
Nagpur, India	0.94 (0.72, 1.23)	1.36 (1.09, 1.71)
Pakistan	0.89 (0.80, 0.99)	0.93 (0.82, 1.07)
Argentina	1.60 (0.99, 2.58)	1.06 (0.54, 2.09)
Guatemala	0.74 (0.68, 0.81)	0.88 (0.73, 1.06)

Relative Risk (RR) for 28-day neonatal mortality comparing intervention to control groups by research site. RR with corresponding 95 % CIs and *p*-values were calculated from generalized linear models accounting for the cluster-level variance and adjusted for randomization strata. For Guatemala we excluded data from entire strata that included the cluster that could not participate due to security concerns

Table 2 presents the percent of births that were <5th percentile and the neonatal mortality rates in the pretrial and trial periods. The percent of neonates classified as <5th percentile was slightly lower in the intervention clusters prior to the trial but became slightly higher during the trial. The overall neonatal mortality was similar in the intervention and control clusters prior to the trial (not surprising since the clusters were randomized in part based on the pretrial neonatal mortality rates). During the trial, the overall neonatal mortality was lower in the intervention clusters, but not significantly so (21.7/1000 vs 26.1/1000, RR 0.88 (0.73, 1.06), *p* = 0.1875).

**Table 2:** Pretrial and trial data by group and period

Characteristic	Pre trial		Trial period, *N* (Rate/1000)		
	Intervention	Control	Intervention	Control	RR (95 % CI), *p*-value
Babies	2,053	2,321	3,800	3,978	--
Live births	2,007 (97.8)	2,271 (97.8)	3,725 (98.0)	3,905 (98.2)	--
<5th percentile live births (% of live births)	77 (3.8)	105 (4.6)	197 (5.3)	166 (4.3)	--
Neonatal death 28 days^a^					
Overall	59 (29.7)	68 (29.9)	81 (21.7)	102 (26.1)	0.88 (0.73, 1.06), *p* = 0.1875
<5th percentile	22 (285.7)	23 (219.0)	36 (182.7)	39 (234.9)	0.74 (0.68, 0.81), *p* = <.0001

^a^The denominator for neonatal mortality is all live births. Of the 2,007 live births in the intervention group during the pre-trial period 21 missing 28-day status are excluded from the denominator

We also compared the <5th percentile neonatal mortality in the intervention and control clusters in the period before the trial to the results during the trial. In the control clusters the neonatal mortality in the <5^th^ percentile infants increased from 219/1000 live births to 235/1000 live births, while in the intervention clusters the neonatal mortality in the <5^th^ percentile infants fell from 286/1000 to 183/1000. Therefore, neonatal mortality in the <5^th^ percentile infants decreased substantially from the pretrial period to the trial period in the intervention clusters, but actually increased slightly in the control clusters. And importantly, the significant difference in neonatal mortality in the <5th percentile infants between the intervention and control clusters during the trial was not due to a lower neonatal mortality in the intervention clusters prior to the trial.

The use of ACS in the intervention and control clusters in the overall population and in the <5th percentile births during the ACT trial was compared. In the intervention clusters, the overall use of ACS was 10.2 % compared to 1.0 % in the control clusters. For the <5th percentile infants, in the intervention clusters, 49.1 % received ACS compared to 13.8 % in the control clusters, nearly a four-fold increase (data not shown).

We evaluated the characteristics of the women in the intervention and control clusters during the trial (Table 3). While similar percentages of women in both groups had no education, more women in the intervention clusters had secondary and university educations. Parity and age distributions appeared similar in the intervention compared to the control clusters. The rate of multiple births was less than 1 % in both groups, but was higher in the intervention clusters.

**Table 3:** Maternal characteristics by intervention and control group

Characteristic	Trial period	
	Intervention	Control
Women, *N*	3,766	3,960
Maternal age, *N* (%)	3,765	3,960
<20	600 (15.9)	639 (16.1)
20–35	2,797 (74.3)	2,888 (72.9)
>35	368 (9.8)	432 (10.9)
Maternal education, *N* (%)	3,766	3,960
No formal education	720 (19.1)	811 (20.5)
Primary	2,239 (59.5)	2,604 (65.8)
Secondary	756 (20.1)	533 (13.5)
University +	51 (1.4)	12 (0.3)
Parity, *N* (%)	3,765	3,960
0	1,122 (29.8)	1,085 (27.4)
1	824 (21.9)	839 (21.2)
2 or more	1,819 (48.3)	2,036 (51.4)
Multiples	3,766	3,960
Yes	34 (0.9)	16 (0.4)
No	3,732 (99.1)	3,944 (99.6)

Next we evaluated some characteristics related to delivery care in the intervention and control clusters during one year prior to the trial and then during the trial period (Tables 4 and 5). First, in both the intervention and control clusters, many of the measures related to the quality of obstetric care clearly improved over time, including the use of hospitals for delivery, physician as the birth attendant and the rate of cesarean delivery [8]. Prior to the trial most of the characteristics evaluated, including hospital delivery, physician attendance and cesarean section usage were either similar between the intervention groups or the higher levels of care favored the control clusters. However, during the trial, these same measurements consistently favored the intervention clusters and suggested that the availability and usage of obstetric care improved to a greater extent in the intervention than in the control clusters. When we evaluated whether the change in care between the pretrial period and the trial period differed between the treatment and control groups, we found large changes for use of vitamins and delivery by a trained attendant that appeared to differ in a meaningful way between the treatment groups; however, the only change that showed a statistically significant difference between treatment groups was vitamin usage (6.46 % (2.02, 10.89 %, *p* = 0.0043) (Table 4). The unadjusted difference of the increase in prevalence in the treatment arm was 13 % more than the control arm (adjusted percent difference of the differences 7.56, 95 % CI −2.09, 18.20 % *p* = 0.1643).

**Table 4:** Prenatal and delivery characteristics by group and period

Characteristic	Pre-trial period		Trial period		Crude Estimate of Change in Prevalence as a Percentage^a^		Crude Estimate of Difference in Change in Prevalence^a^	Adjusted Estimate of Difference in Change in Prevalence (95 % CI), *p*-value^b^
	Intervention	Control	Intervention	Control	Intervention	Control		
Deliveries, *N*	2,041	2,305	3,766	3,960				
At least one antenatal care visit, (%)	98.7	97.3	99.2	99.2	0.5	1.9	−1.4	−1.72 (−4.00, 0.55), *p* = 0.1379
Prenatal vitamins/iron, (%)	85.9	89.7	93.7	91.4	7.8	1.7	6.1	6.46 (2.02, 10.89), *p* = 0.0043
Birth attendant, (%)					26.0	13.0	13.0	7.56 (−3.09, 18.20), *p* = 0.1643
Physician	26.0	32.3	50.6	45.1				
Nurse/Midwife/HW	1.7	0.2	3.1	0.5				
TBA	72.1	67.1	45.7	53.9				
Family/Other	0.2	0.4	0.6	0.6				
Delivery mode, (%)					−11.1	−7.1	−4.0	−2.21 (−8.17, 3.76), *p* = 0.4689
Vaginal	87.9	87.5	76.8	80.4				
C-section	12.1	12.5	23.2	19.6				
BA used new gloves, (%)	98.5	97.6	99.3	99.0	0.8	1.4	−0.6	−0.53 (−2.02, 0.97), *p* = 0.4894

^a^Crude change in prevalence = percentage during the trial period minus percentage during the pretrial period

^b^Test to assess whether the change between the pretrial and trial periods differed by treatment group. *P*-values calculated from generalized linear models accounting for the cluster-level variance and adjusted for randomization strata

**Table 5:** Delivery location characteristics by group and period

Characteristic	Pre trial period		Trial period		Crude Estimate of Change in Prevalence^a^		Crude Estimate of Difference in Change in Prevalence^b^	Adjusted Estimate of Difference in Change in Prevalence (95 % CI), *p*-value^c^
	Intervention	Control	Intervention	Control	Intervention	Control		
Deliveries, *N*	2,041	2,305	3,766	3,960				
Delivery location, (%)								--
Hospital	24.9	29.8	46.8	44.3	21.9	14.5	7.4	
Clinic	2.5	2.3	7.0	1.1	4.5	−1.2	5.7	
Home/Other	72.6	67.9	46.2	54.6	−26.4	−13.3	−13.1	
Birth at facility level (hospital or clinic), (%)	27.4	32.1	53.8	45.4	26.4	13.3	13.1	7.70 (−2.61, 18.01), *p* = 0.1434
Birth at facility with C-section or any neonatal care capabilities^d^, (%)	26.2	31.0	53.3	45.2	27.1	14.2	12.9	7.36 (−3.39, 18.12), *p* = 0.1797
Birth at facility that has C-section capabilities, (%)	26.1	31.0	47.5	40.5	21.4	9.5	11.9	--
Birth at facility that has bag and mask capabilities, (%)	10.7	11.1	43.7	41.9	33.0	30.8	2.2	--
Birth at facility that has oxygen or mechanical ventilation capabilities, (%)	20.8	26.6	47.4	42.6	26.6	16.0	10.6	--

^a^Crude change in prevalence = (percentage during the trial period minus percentage during the pretrial period)

^b^Crude difference in change in prevalence = (percentage during the trial period minus percentage during the pretrial period for the treatment group) minus (percentage during the trial period minus percentage during the pretrial period for control group)

^c^Test to assess whether the change between the pretrial and trial periods differed by treatment group. The adjusted percent difference of the percent differences is the estimate of the difference in changes in probability from pretrial to trial period for the given characteristic in the treatment arm minus changes in probability from the pretrial to trial period for the given characteristic in the control arm. *P*-values were calculated from generalized linear models accounting for the cluster-level variance and adjusted for randomization strata

^d^Neonatal care capabilities include bag and mask, oxygen or mechanical ventilation. Facilities with any of the capabilities are tested for differences between the pretrial and trial periods by treatment group

We also evaluated the capability of the facilities used for deliveries in the intervention and control clusters in the pretrial and trial periods (Table 5). Births in a facility of any kind increased substantially in both the intervention and control clusters, but to a greater extent in the intervention clusters (adjusted difference in change in prevalence of delivery in facility 7.70, 95 % CI −2.61, 18.01, *p* = 0.1434). When the capability of the facilities was evaluated, there were again substantial improvements from the pretrial to the trial period in births at facilities with cesarean section capabilities, bag and mask capabilities for neonatal resuscitation and oxygen. When the percent of births in facilities with any of those capabilities was evaluated, there was a substantial increase in these characteristics from the pretrial to the trial period, but the increases were greater in the intervention compared to the control clusters but the difference was not statistically significant (adjusted difference in change in prevalence 7.36, 95 % CI −3.39, 18.12, *p* = 0.1797).

We compared the overall neonatal mortality rates by location of birth (home or facility) in the pretrial compared to the trial period in the intervention and control clusters (Table 6). In the home births, the mortality rates were all between 21/1000 and 28/1000 in the pretrial and trial periods. There was however, a small decrease in the neonatal mortality rate in the control clusters from the pre-trial to the trial period. (28/1000 to 23/1000). In the facility births in the pretrial period the overall neonatal mortality was considerably higher in the intervention compared to the control clusters. However, there was a substantial decrease in the neonatal mortality rate in the facility births in the intervention clusters such that during the trial the mortality rates in the intervention clusters was 21.9/1000 births compared to 29.8 in the control clusters. Furthermore, when we evaluated the rates of neonatal mortality in the intervention compared to the control clusters in facilities able to provide cesarean section, or any neonatal care capability (bag and mask resuscitation, oxygen or mechanical ventilation), the difference in the neonatal mortality rate was substantial (22.1/1000 vs 30.0/1000 live births).

**Table 6:** Mortality rates by delivery location and period

Characteristic^a^	Pre-trial period		Trial period	
	Intervention	Control	Intervention	Control
Live births, *N*	2,007	2,271	3,725	3,905
Neonatal deaths <28d, *N* (Rate/1000) in home birth	21.4	27.8	21.6	23.0
Neonatal deaths <28d, *N* (Rate/1000) in facility (clinic or hospital)	52.0	34.4	21.9	29.8
Neonatal deaths <28d, *N* (Rate/1000) in facility with C-section or any neonatal care capabilities^b^	52.4	32.7	22.1	30.0

*Note*: The table provides rates for neonatal mortality by location of delivery. The event counts within clusters were too small to get reliable estimates of relative risk for treatment versus control groups, or for probability differences for changes between the pretrial and trial periods by treatment group

^a^The denominator for neonatal mortality is all live births. Of the 2,007 live births in the intervention group during the pre-trial period 21 are missing 28-day status and are excluded from the denominator

^b^Neonatal care capabilities include bag and mask, oxygen or mechanical ventilation

In Table 7 we compared the neonatal mortality within each of the birth weight groups in the intervention and control clusters during the trial period. Substantial differences in mortality occurred in both the 1500–2499 g group and in the ≥2500 g birth weight groups in the intervention compared to the control clusters. Since the 5^th^ percentile birth weight in Guatemala was 2267 g, and since most of the births <2500 g weighed 1500 to 2499 g, there is reasonably good concordance between the results by birth weight percentiles and by specific birth weight groups.

**Table 7:** Mortality rates by birth weight in intervention and control clusters during the trial period among live births

Characteristics	Trial period	
	Intervention	Control
Live births, *N*	3,725	3,905
Mortality for births <1000 g		
Births <1000 g, *N*	10	7
Neonatal deaths, *N*	9	7
Neonatal mortality < 28 days, *n* (rate/1000)	900	1000
Mortality for births 1000–1499 g		
Births 1000–1499 g, *N*	22	20
Neonatal deaths, *N*	14	10
Neonatal mortality < 28 days, *n* (rate/1000)	636	500
Mortality rates for births 1500–2499 g		
Births 1500–2499 g, *N*	487	454
Neonatal deaths, *N*	20	33
Neonatal mortality < 28 days, *n* (rate/1000)	41.1	72.7
Mortality rates for births ≥ 2500 g		
Births ≥ 2500 g, *N*	3,206	3,424
Neonatal deaths, *N*	38	52
Neonatal mortality < 28 days, *n* (rate/1000)	11.9	15.2

*Note*: The table provides frequencies and rates for neonatal mortality by birth weight group. The event counts within clusters are too small to get reliable estimates of relative risk for treatment versus control groups

Finally, we evaluated measures of maternal and neonatal infection during the trial in the intervention and control clusters in the overall population and in the <5th percentile births (Table 8). There were essentially no differences in the maternal infection measure between the intervention and control clusters in the entire population or in the mothers of the <5^th^ percentile babies. The measure of neonatal infection among the intervention group when compared to the control group was slightly greater in both the entire population of newborns, and in the <5^th^ percentile babies but neither difference was significant.

**Table 8:** Indicators of infection in the intervention and control clusters during the trial period

Characteristics	Trial period		
	Intervention	Control	RR (95 % CI), *p*-value^a^
Suspected maternal infection			
Overall *N*,(%)	46/3,766 (1.2)	53/3,960 (1.3)	N/A
<5th percentile *N*,(%)	6/181 (3.3)	5/158 (3.2)	N/A
Possible severe bacterial infection (pSBI)			
Overall *N*,(%)	508/3,725 (13.6)	460/3,904 (11.8)	1.13 (0.88–1.45), *p* = 0.3371
<5th percentile *N*,(%)	68/197 (34.5)	54/166 (32.5)	1.06 (0.82–1.36), *p* = 0.6530

^a^RR with corresponding 95 % CIs and *p*-values were calculated from generalized linear models accounting for the cluster-level variance and adjusted for randomization strata. For maternal infection, the event counts within clusters were too small to get reliable estimates of relative risk

## Discussion

First, we emphasize that because this is a secondary analysis from a single site of a multisite trial, the results are exploratory in nature. We are presenting these data as a case study, to explore factors related to the mothers, the infants and the quality of care, including the use of ACS. This analysis is an attempt to understand the outcomes in Chimaltenango, Guatemala, which showed benefit in the intervention clusters in the targeted <5^th^ birth weight percentile, differing from the overall findings of the ACT trial. The intervention aimed at improving identification of women likely to deliver preterm within 7 days and in the appropriate gestational age range and to facilitate appropriate use of antenatal corticosteroids for those women. The results showed a significant reduction in neonatal mortality in the <5^th^ percentile infants in the Guatemala site.

During the trial, in the intervention clusters, irrespective of where delivery occurred (facility or home), the neonatal mortality rates were lower than deliveries in control clusters. However, the largest differences were observed in facility births. The reduction in neonatal mortality in the intervention clusters occurred in babies over 1500 g and was greatest and significant in the < 5th percentile infants.

We explored various factors that could explain these differences. The difference in neonatal mortality between the intervention and control clusters was not explained by differences in neonatal mortality in the pretrial period. We found no large differences in the populations of the intervention and control clusters; the proportion of births below the 5^th^ percentile and the characteristics of mothers were similar overall in both groups during the trial.

During the ACT trial, the quantity of prenatal care did not appear to be different between intervention and control clusters, although the use of vitamins was increased in the intervention clusters. However, care at delivery appeared better in intervention clusters including delivery at a hospital or clinic, delivery by a physician and use of cesarean section, although the differences were not different statistically. ACS use in the intervention clusters was significantly higher than in the control clusters, 49.1 % compared to 13.8 %. Evidence of maternal or neonatal infection did not appear to be different between the two groups.

## Conclusions

In conclusion, whether the improvement in care in the intervention clusters compared to the control clusters was due to the multipronged ACT intervention during the ACT Trial or occurred independently is unknown. In addition, the care available to pregnant women and newborns in the Chimaltenango hospital appears to be at a higher level than that available in many of the other trial sites and in some respects approaches that seen in high-income countries where most trials of ACS have shown benefits. If the Guatemalan results did not occur by chance, we suspect that the combination of improved care and greater ACS use may account for the observed difference in the <5^th^ percentile neonatal mortality between the intervention and control clusters.
